# Antimicrobial resistance of avian pathogenic *Escherichia coli* isolated from broiler, layer, and breeder chickens

**DOI:** 10.14202/vetworld.2024.480-499

**Published:** 2024-02-29

**Authors:** Rebanta K. Bhattarai, Hom B. Basnet, Ishwari P. Dhakal, Bhuminand Devkota

**Affiliations:** 1 Department of Veterinary Microbiology and Parasitology, Faculty of Animal Science, Veterinary Science and Fisheries, Agriculture and Forestry University, Nepal; 2 Department of Medicine and Public Health, Faculty of Animal Science, Veterinary Science and Fisheries, Agriculture and Forestry University, Nepal; 3 Department of Theriogenology, Faculty of Animal Science, Veterinary Science and Fisheries, Agriculture and Forestry University, Nepal

**Keywords:** antibiotic resistance gene, multiplex PCR, colibacillosis, multiple antibiotic resistance index, *mcr1*, commercial chicken

## Abstract

**Background and Aim:**

Antimicrobials are extensively used in poultry production for growth promotion as well as for the treatment and control of diseases, including avian pathogenic *Escherichia coli* (APEC). Poor selection, overuse, and misuse of antimicrobial agents may promote the emergence and dissemination of antimicrobial resistance (AMR) in APEC. This study aimed to assess antimicrobial susceptibility patterns and detect antibiotic resistance genes (ARGs) in APEC isolated from clinical cases of colibacillosis in commercial broiler, layer, and breeder chickens.

**Materials and Methods:**

A total of 487 APEC were isolated from 539 across 300 poultry farms in various regions of Nepal. Antimicrobial susceptibility patterns was determined using the Kirby-Bauer disk diffusion and broth microdilution methods. The index of AMR, such as multiple antibiotic resistance (MAR) index, resistance score (R-score), and multidrug resistance (MDR) profile, were determined. Polymerase chain reaction was employed to detect multiple ARGs and correlations between phenotypic and genotypic resistance were analyzed.

**Results:**

The prevalence of APEC was 91% (487/539). All of these isolates were found resistant to at least one antimicrobial agent, and 41.7% of the isolates were resistant against 8–9 different antimicrobials. The antibiogram of APEC isolates overall showed the highest resistance against ampicillin (99.4%), whereas the highest intermediate resistance was observed in enrofloxacin (92%). The MAR index and R-score showed significant differences between broiler and layers, as well as between broiler breeder and layers. The number of isolates that were resistant to at least one agent in three or more antimicrobial categories tested was 446 (91.6%) and were classified as MDR-positive isolates. The ARGs were identified in 439 (90.1%) APEC isolates, including the most detected mobilized colistin resistanc*e* (*mcr*1) which was detected in the highest (52.6%) isolates. Overall, resistance gene of beta-lactam (*blaTEM*), *mcr1*, resistance gene of sulphonamide (*sul1*) and resistance gene of tetracycline (*tetB*) (in broiler), were detected in significantly higher than other tested genes (p < 0.001). When examining the pair-wise correlations, a significant phenotype-phenotype correlation (p < 0.001) was observed between levofloxacin and ciprofloxacin, chloramphenicol and tetracycline with doxycycline. Similarly, a significant phenotype-genotype correlation (p < 0.001) was observed between chloramphenicol and the *tetB*, and colistin with *blaTEM* and resistance gene of quinolone (*qnrA*).

**Conclusion:**

In this study, the current state of APEC AMR in commercial chickens is revealed for the first time in Nepal. We deciphered the complex nature of AMR in APEC populations. This information of molecular surveillance is useful to combat AMR in APEC and to contribute to manage APEC associated diseases and develop policies and guidelines to enhance the commercial chicken production.

## Introduction

Avian pathogenic *Escherichia coli* (APEC) causes colibacillosis in avian species, resulting in extraintestinal lesions such as air-sacculitis, pericarditis, peritonitis, salpingitis, synovitis, osteomyelitis, and yolk sac infections [1]. Colibacillosis leads to substantial economic losses in the poultry industry [2] due to high mortality and low production efficiency in affected birds [3]. The pathogen is also known for its zoonotic potential [4] associated with the high public health threat [5]. Therefore, effective control strategies for APEC are crucial in modern poultry production and the utilization of poultry product. Antimicrobial resistance (AMR) has been extensively studied in humans, livestock, and poultry products. AMR is a global health challenge that occurs when pathogens evolve over time and no longer respond to medications, making infections increasingly difficult to treat [6]. The AMR issue arises from the improper selection, overuse, and misuse of critical antimicrobial agents in human and veterinary medicine [7]. Notably, there is a lack of AMR surveillance in the veterinary industry in developing countries, including Nepal, coupled with inadequate veterinary medication regulation and guidelines [8]. The detection of antibiotic resistance genes (ARGs) is pivotal in controlling avian colibacillosis with multidrug resistance (MDR) APEC [9]. In Nepal, 14 antibiotic types are available for veterinary use, among which tetracycline (T), enrofloxacin (ENF), neomycin (NE), doxycycline (DXT), levofloxacin (LEV), colistin (CO), and tylosin are primarily used for treating colibacillosis [9]. Indices such as multiple antibiotic resistance (MAR) index, resistance-score (R-score), and MDR profile serve as markers for the spread of AMR in bacterial populations. These indices are rapid, easy, cost-effective, and valid methods for comparing AMR from phenotypic antimicrobial susceptibility test (AST) results. MAR indices exceeding 0.2 suggest antibiotic usage in sources with high intensity and/or in large quantities [10], creating selective pressure in APEC strains [11]. This pressure contributes to the emergence of highly challenging MDR organisms on farms [12] raising concerns about their transmission to humans after consuming contaminated food of animal origin [13]. MDR APEC acting as a reservoir of resistance genes, has the ability to transfer these genes to other pathogens in hosts as well as the human intestinal system [14], posing serious concerns about antibiotics use in poultry [15]. A significant proportion of AMR APEC is recovered from chickens compared with other animals [16] having similar virulence properties [17], particularly in a context of the national poultry population significantly expanding (85 million in 2021 compared with 21 million in 2001) [18]. *E. coli* holds the top position among the twelve bacterial families considered the greatest threat to human and animal health [19]. MDR is defined as AMR shown by a microorganism to at least one antimicrobial drug in three or more antimicrobial categories [20]. It is predicted that antimicrobial usage in chickens will increase by a 129% increase in Asia by 2030 [21]. In Nepal, 46% of veterinary drugs are sold without prescription, and 12% are based on farmers’ demand [22]. This is linked to population growth, changing food habits with increased chicken consumption, and poor antibiotic stewardship [23]. Moreover, these compounds are commonly used as growth promoters in poultry farms [19, 24]. The challenge of MDR in APEC strains is particularly in lower to middle-income nations, like Nepal, due to inadequate diagnostic capabilities.

Phenotypic AMR alone is insufficient to determine the extent of the AMR problems. Genotypically, AMR is indicted by the presence of ARGs in individual APEC strains. Information on the identification of AMR profiles, both phenotypic and genotypic ARGs, and the correlation of phenotypic and genotypic resistance in APEC from commercial poultry is lacking in Nepal. The consumption of chicken meat contaminated with AMR pathogens is a serious hazard, as the spread of APEC among different chicken types and other contaminated food sources may facilitate the transmission of it to other animal species and humans [25]. Moreover, MDR organisms can also spread vertically through contaminated eggs from sick breeders, contaminating commercial meat and eggs [26]. This highlights the need for screening AMR and monitoring proper antimicrobial use to effectively control the spread of AMR pathogens. The spread of MDR pathogens may be heightened in Nepal due to poor biosecurity, high antibiotic use [9], and horizontal gene transfer [22]. Therefore, closely monitoring the landscape of antibiotic resistance in Nepalese poultry industry is crucial.

To address the knowledge gap on AMR, MDR, and ARGs burden in commercial chickens in Nepal, we aimed to detect antimicrobial susceptibility patterns and identify ARGs in colibacillosis-confirmed broiler, layer, and breeder chickens from different parts of Nepal. This study focuses on the AMR profile and detection of ARGs, which has significant implications for controlling AMR pathogens. Ultimately supporting improvement in poultry and human health.

## Materials and Methods

### Ethical approval

Ethical approval was not necessary for this study. Samples were collected during the necropsy of the chickens. However, samples were collected aseptically in accordance with established collection methods.

### Study period and location

This study was conducted from September 2019 to October 2020 in Chitwan, Dang, Rupandehi, Makawanpur, Palpa, Nawalpur, and Arghakhachi districts of Nepal.

### Sample collection and bacterial isolation and identification

A total of 487 APEC isolates were identified from 539 dead broiler, layer, and breeder chickens affected with colibacillosis. These isolates were collected from aforementioned locations that are known as poultry pockets of Nepal. The methods of diagnosing colibacillosis, as well as isolation and identification of *E. coli*, DNA extraction and quantification, and gel electrophoresis have been described by Bhattarai *et al*. [27]. A sample pool was collected from the heart blood, liver, and bone marrow of each flock of dead commercial chickens with pathological lesions of colibacillosis. These samples were streaked onto MacConkey and Eosin Methylene Blue agar plates and incubated at 37°C for 24 h. *E. coli* isolates were confirmed using standard biochemical and bacteriological methods [28], which was performed at Department of Microbiology and Parasitology, Faculty of Animal Science, Veterinary Science and Fisheries (FAVF), Agriculture and Forestry University (AFU), Rampur, Chitwan, Nepal. Genomic DNA was extracted from *E. coli* isolates using the boiling methods as previously described [29].

### AST

ASTs were performed using both the disk diffusion method and determination of minimum inhibitory concentration (MIC) by broth microdilution (BMD). The Kirby-Bauer disk diffusion method was used for the determination of antimicrobial susceptibility [30]. Commercial antimicrobial-impregnated disks (Mast Group, UK) of fourteen different veterinary critically important antimicrobial agents (VCIA) [31] representing eight different antimicrobial classes (Supplementary material) were used to assess antimicrobial susceptibility. For the preparation of inoculum and application of the disk on the surface of the Muller Hinton agar (M173, HiMedia, India), the procedure described by Bauer [30] was followed. The HiAntibiotic Zone Scale (HiMedia) facilitated the measurement of the diameters of the zones of complete inhibition, including the disk diameter, in millimeters. The defined Clinical and Laboratory Standards Institute classification based on the resistance breakpoint was used to categorize isolates as resistant (R), intermediate susceptible (I), and susceptible (S) [32].

The MAR index (the ratio of the number of antibiotics to which an organism is R [i.e. “a” herein] to the total number of antibiotics to which the organism is exposed [i.e. “b”]) was calculated as a/b [10]. APEC strains with a MAR index ≥0.2 were considered to originate from a source with a high risk of contamination where several antibiotics are used. For each APEC isolate, the R-score was defined as the number of antibiotics against which the isolate exhibited I or R. The R-scores of 0.5 and 1 were attributed to isolates exhibiting I or R, respectively, to a specific antibiotic [33]. Isolates that were R to at least one agent in ≥3 antimicrobial categories were categorized as MDR-positive as per the definition by Magiorakos *et al*. [20]. To assess the antimicrobial susceptibility against CO, inoculum preparation and the MIC test using BMD was conducted [34]. A lab-grade 0.8 mg CO tablet (TAB/CO0.8, ADATAB®, Mast Group) dissolved in 100 mL nuclease-free water (NFW) produced a breakpoint concentration of 8 mg/L. A stock solution of 800 mg/L (equivalent to 800 μg/mL) was prepared by mixing 1 mL of NFW with the CO tablet. Next, 840 μL was added to 160 μL of stock solution to create a working solution with a concentration of 128 μg/mL. The CO concentration range was set, which is between 0.125 μg/mL and 128 μg/mL. Inoculum preparation, dilution, and determination of BMD endpoints were performed according to National Centre for Disease Control [35]. Reading and interpretation of CO-S and CO-R APEC isolates were based on MICs ≤2 μg/mL and >2 μg/mL, respectively, following the European Committee on Antimicrobial Susceptibility Testing (EUCAST) methodology [34] with *E. coli* strain American Type Culture Collection 25922 used as the quality control organism in all tests.

### Detection of ARGs by multiplex polymerase chain reaction (PCR)

Following primer compatibility and product size consideration, a set of specific primers (Sigma-Aldrich, Germany) was grouped into single multiplexes (Table-1) [36–39]. Initially, using template DNA from suitable positive and negative control strains in single PCR experiments for ARGs, we verified primers for traditional multiplex PCR to identify resistance genes of beta-lactams (*blaTEM*), resistance genes of sulphonamide (*sul1*), resistance genes of quinolone (*qnrA*), resistance genes of tetracycline (*tetB*), resistance genes of chloramphenicol (*cat1*), resistance genes of erythromycin (*ereA*), mobilized colistin resistance genes (*mcr1*), and resistance genes of gentamicin (*aac(3)–IV*). Once the primers were confirmed, the *E. coli* isolates were examined for the presence of eight ARGs using multiplex PCR. This evaluation was performed on DNA samples extracted from 487 APEC isolates, adhering to the revised protocol [40].

**Table-1:** Primer sequences used multiplex PCR for the identification of ARGs in APEC isolates.

Target gene	Primer sequence (5´ – 3´)	Amplicon size (bp)	Reference
*blaTEM*	F – GAGTATTCAACATTTTCGT	857	[36]
	R – ACCAATGCTTAATCAGTGA		
*sul1*	F – TTCGGCATTCTGAATCTCAC	822	[36]
	R – ATGATCTAACCCTCGGTCTC		
*cat1*	F – AGTTGCTCAATGTACCTATAACC	547	[36]
	R – TTGTAATTCATTAAGCATTCTGCC		
*ereA*	F – GCCGGTGCTCATGAACTTGAG	419	[36]
	R – CGACTCTATTCGATCAGAGGC		
*aac(3)-IV*	F – CTTCAGGATGGCAAGTTGGT	286	[36]
	R - TCATCTCGTTCTCCGCTCAT		
*qnrA*	F – GGGTATGGATATTATTGATAAAG	670	[37]
	R – CTAATCCGGCAGCACTATTTA		
*tetB*	F – CCTCAGCTTCTCAACGCGTG	634	[38]
	R – GCACCTTGCTGATGACTCTT		
*mcr1*	F – CGGTCAGTCCGTTTGTTC	309	[39]
	R – CTTGGTCGGTCTGTAGGG		

PCR=Polymerase chain reaction, APEC=Avian pathogenic *E. coli*, ARGs=Antibiotic resistance gene, F=Forward, R=Reverse, *blaTEM*=Resistant gene of beta-lactam, *sul1*=Resistant gene of sulphonamides *ereA=R*esistant gene of erythromycin*, aac(3)-IV=*Resistant gene of gentamicin, *qnrA=*Resistant gene of quinolone

A final volume of 36 μL reaction mixtures contained 18 μL of platinum green hot start PCR master mix (2×) (13001014, Invitrogen, USA), 200 ng of genomic DNA, 10 pmol of each forward and reverse primer from eight different primers (Sigma-Aldrich), and NFW. For multiplex PCR, the following conditions were adjusted on the Thermal Cycler (Bio-Rad T100TM, USA): Initial denaturation at 94°C for 5 min, followed by 30 cycles of denaturation at 94°C for 30 s, annealing at 58°C for 30 s, and extension at 68°C for 10 min, and a final cycle of amplification at 72°C for 10 min and holding at 4°C for infinity [40].

### Statistical analysis

For the evaluation of AST, MDR, and ARGs, percentage values were used. We used the Chi-square test for relatedness and one-way analysis of variance to evaluate further the relationships between the various chicken types and the distribution of ARGs. We also used these tests to estimate the overall differences between the frequencies of associated ARGs against APEC isolates. Because the sample size of each chicken type was different, we performed Levene’s test for homogeneity of variance. Variables with equal variance were compared using the Bonferroni test, while those with unequal variance underwent mean comparison using Dunnett’s test. Significant differences between the MAR index and R-score with types of chicken were determined by the Kruskal-Wallis test. Furthermore, we used Spearman’s rank correlation coefficients (r_s_) to determine the association between pairs of AMR, ARGs, and phenotypic versus genotypic resistance isolated from different types of chickens. We analyzed the data using IBM Statistical Package for the Social Sciences Version 25.0 (IBM Corp., New York, USA). From the AST data, the MAR index, MDR, and R-score were computed using Excel (Supplementary material). A p ≤ 0.05 was considered statistically significant. The calculated p-value is mentioned in three digits after the decimal, and the rest of the p-values are mentioned in <0.001. While performing statistical analysis, we regarded I isolates (i.e., indicating an uncertain therapeutic effect) as R.

## Results

### Antimicrobial susceptibility pattern

The highest mean zone of inhibition was observed with amikacin (AK) (18.3 ± 4.1 mm) followed by chloramphenicol (C) (14.8 ± 8.4 mm), gentamicin (GEN) (14 ± 6.4 mm), LEV (12.1 ± 7.2 mm), NE (12.1 ± 4 mm), ciprofloxacin (CIP) (12 ± 8.9 mm), cotrimoxazole (TS) (8.5 ± 10 mm), DXT (7.8 ± 5.4 mm), ENF (7.5 ± 8.5 mm), and the lowest was seen with tetracycline (3.9 ± 7.2 mm) (Table-2). The mean zones of inhibition by AK, C, TS, CIP, ceftriaxone (CRO), GEN, azithromycin (ATH), and LEV were significantly different among the various chicken types, indicating distinct types of resistance among them. For CO, the mean MIC was found to be 0.98 μg for overall chicken (Table-2), and this value did not show significant differences among chicken types.

**Table-2:** Mean zone of inhibition (in mm) of APEC strains among different types of chickens (n = 487).

Antimicrobials	Broiler	Broiler breeder	Layer	Layer breeder	Overall mean	F-test	p-value
Aminoglycosides							
NE, 10 μg	11.9	12.9	11.7	12.0	12.1	1.737	0.159
GEN, 10 μg	13.3^a^	14.8^ab^	14.8^ab^	17.2^b^	14.0	3.984*	0.008
AK, 30 μg	17.9^a^	19.9^b^	17.5^a^	17.7^a^	18.3	7.822**	<0.001
Penicillin							
AP, 10 μg	2.8	2.7	3.2	3.1	2.8	0.253	0.859
Extended-spectrum cephalosporin							
CRO, 5 μg	22.3^a^	22.4^b^	22.2^ab^	13.2^a^	21.9	13.366**	<0.001
Sulphonamide							
TS, 25 μg	6.7^a^	12.3^a^	9.6^b^	8.3^a^	8.5	9.258**	<0.001
Tetracyclines							
DXT, 30 μg	8.1	6.7	8.2	8.2	7.8	1.952	0.120
T, 30 μg	3.9^ab^	3.3^ab^	5.4^b^	1.9^a^	3.9	1.971	0.117
Macrolides							
ATH, 15 μg	18.8	19.1	19.9	19.6	19.1	3.688*	0.012
Phenicol							
C, 30 μg	15.2^b^	11.3^a^	17.6^b^	17.7^b^	14.8	11.091**	<0.001
Fluoroquinolones							
CIP, 5 μg	10.6^a^	12.1^ab^	16.1^c^	14.5^bc^	12.0	9.509**	<0.001
ENF, 30 μg	7.3^ab^	6.8^ab^	9.5^b^	5.9^a^	7.5	1.991	0.115
LEV, 5 μg	11.5	12.1	14.2	12.1	12.1	2.965*	0.032
Polymyxin							
CO, 0.8 mg (MIC value)	1.01	0.98	0.98	0.61	0.98	0.994	0.395

* and

** indicates significant (p ≤ 0.05) and highly significant (p ≤ 0.01), respectively. different superscript in the same row means significant difference. NE=Neomycin, GEN=Gentamicin, AK=Amikacin, AP=Ampicillin, CRO=Ceftriaxone, TS=Cotrimoxazole, DXT=Doxycycline, T=Tetracyclines, ATH=Azithromycin, C=Chloramphenicol, CIP=Ciprofloxacin, ENF=Enrofloxacin, LEV=Levofloxacin, CO=Colistin, MIC=Minimum inhibitory concentration, APEC=Avian pathogenic *E. coli*

**Figure-1:**
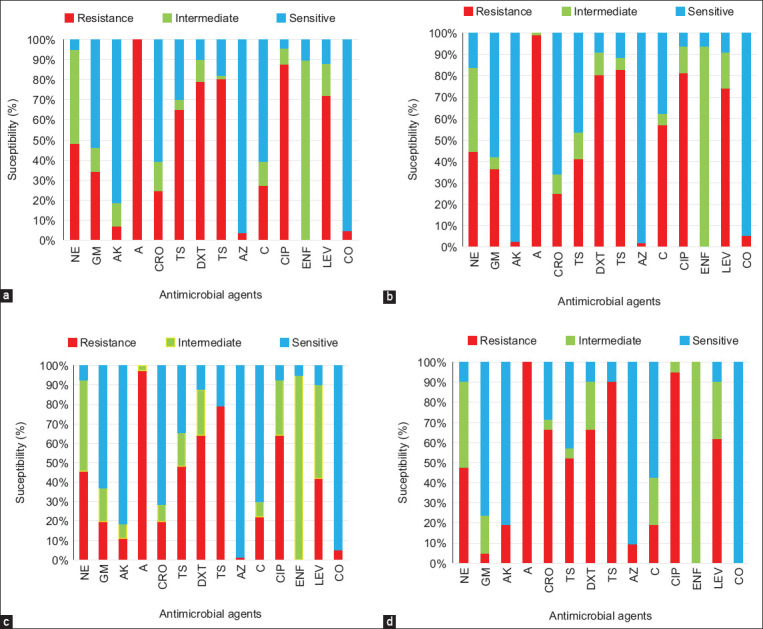
Frequency of antimicrobial susceptibility pattern in avian pathogenic *Escherichia coli* isolates. (a) broiler (n = 273). (b) broiler breeder (n = 112). (c) layer (n = 81). (d) layer breeder (n = 21). NE=Neomycin, GEN=Gentamicin, AK=Amikacin, AP=Ampicillin, CRO=Ceftriaxone, TS=Cotrimoxazole, DXT=Doxycycline, T=Tetracycline, ATH=Azithromycin, C=Chloramphenicol, CIP=Ciprofloxacin, ENF=Enrofloxacin, LEV=Levofloxacin, CO=Colistin.

**Figure-2:**
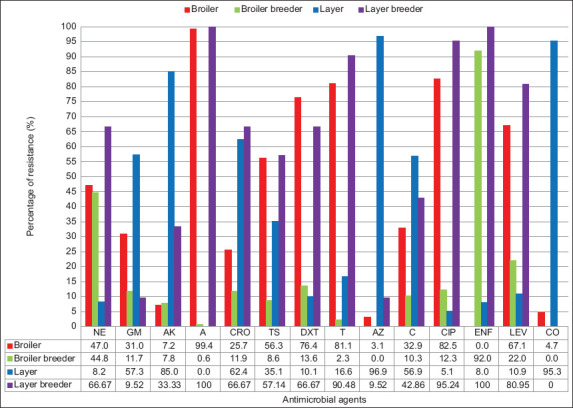
Overall, frequency of antibiotics resistance pattern among different types of chickens (broiler, broiler breeder, layer and layer breeder). NE=Neomycin, GEN=Gentamicin, AK=Amikacin, AP=Ampicillin, CRO=Ceftriaxone, TS=Cotrimoxazole, DXT=Doxycycline, T=Tetracycline, ATH=Azithromycin, C=Chloramphenicol, CIP=Ciprofloxacin, ENF=Enrofloxacin, LEV=Levofloxacin, CO=Colistin.

All the APEC isolates from broilers, broiler breeders, and layer breeders were R to ampicillin (AP). Likewise, 100% of the isolates from layer breeders were R to ENF (Figures-1a–d). Aggregately, the highest resistance was observed against AP (99.4%) followed by CIP (82.5%), T (81.1%), DXT (76.4%), and LEV (67.1%). The highest I was observed against ENF (92%) and NE (44.8%) (Table-3). The percentage of resistance was measured among antimicrobials with I combined with R to AP, CIP, ENF, NE, DXT, LEV, and T, resulting in 83%–100% R (Figure-2).

**Table-3:** *In vitro* antimicrobial susceptibility pattern in APEC isolates from commercial chickens (n = 487: broiler [n = 273], broiler breeder [n = 112], layer [n = 81], and layer breeder [n = 21]).

Class and antimicrobial agents	Susceptible % (No.)	Intermediate % (No.)	Resistance % (No.)
Aminoglycosides			
NE, 10 μg	8.2 (40)	44.8 (218)	47 (229)
GEN, 10 μg	57.3 (279)	11.7 (57)	31 (151)
AK, 30 μg	85 (414)	7.8 (38)	7.2 (35)
Penicillin			
AP, 10 μg	0	0.6 (3)	99.4 (484)
Extended-spectrum cephalosporin			
CRO, 30 μg	62.4 (304)	11.9 (58)	25.7 (125)
Sulphonamide			
TS 250 μg	35.1 (171)	8.6 (42)	56.3 (274)
Tetracyclines			
DXT, 30 μg	10.1 (49)	13.6 (66)	76.4 (372)
T, 30 μg	16.6 (81)	2.3 (11)	81.1 (395)
Macrolides			
ATH, 15 μg	96.9 (472)	0	3.1 (15)
Phenicol			
C, 30 μg	56.9 (277)	10.3 (50)	32.9 (160)
Fluoroquinolones			
CIP, 5 μg	5.1 (25)	12.3 (60)	82.5 (402)
ENF, 10 μg	8 (39)	92 (448)	0
LEV, 5 μg	10.9 (53)	22 (107)	67.1 (327)
Polymyxin			
CO, 0.8 mg	95.3 (464)	0	4.7 (23)
Chi-square	423.28	271.98	
p-value	<0.001	<0.001	

*and **indicates significant (p ≤ 0.05) and highly significant (p ≤ 0.01), respectively, APEC=Avian pathogenic *Escherichia coli*, NE=Neomycin, GEN=Gentamicin, AK=Amikacin, AP=Ampicillin, CRO=Ceftriaxone, TS=Cotrimoxazole, DXT=Doxycycline, T=Tetracycline, ATH=Azithromycin, C=Chloramphenicol, CIP=Ciprofloxacin, ENF=Enrofloxacin, LEV=Levofloxacin, CO=Colistin

Individually, CO was S in 100% isolates in layer breeders (Figure-1b). Collectively, the most S antimicrobials were ATH (96.9%), followed by CO (95.3%), AK (85%), CRO (62.4%), GEN (57.3%), and C (56.9%) (Table-3). The APEC strains were highly significantly R/S against the tested antimicrobials (p < 0.001, Table-3).

### Antimicrobial resistance pattern, MAR index, R-score, and MDR of APEC isolates

**Figure-3:**
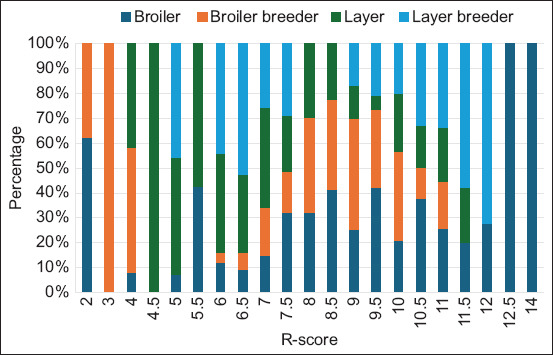
Resistance score of antibiotics resistance pattern of different types of chickens.

We observed 12 antibiotic resistance patterns in APEC isolates in different chicken types (Table-4). The overall pattern of resistance against nine antibiotics was the most prevalent (22.8%, n = 111), followed by eight antibiotics (18.9%, n = 92). Typically, one isolate from the broiler showed resistance to all (n = 14) tested antimicrobials (Table-4). Most of the APEC isolates were with R-scores between 7 and 10 in all chicken types (Figure-3) indicating high R-score.

**Table-4:** Antimicrobial resistant patterns and MAR index of APEC isolates (n = 487) in different types of chickens.

Antimicrobial resistant patterns*	MAR index	Number of resistant isolates				

		Broiler	Broiler breeder	Layer	Layer breeder	Overall
2	0.1	4 (0.8)	1 (0.2)	0	0	5 (1)
3	0.2	1 (0.2)	1 (0.2)	2 (0.4)	0	4 (0.8)
4	0.3	2 (0.4)	5 (1)	4 (0.8)	1 (0.2)	12 (2.5)
5	0.4	9 (1.8)	0	12 (2.5)	1 (0.2)	22 (4.5)
6	0.4	18 (3.7)	5 (1)	13 (2.7)	4 (0.8)	40 (8.2)
7	0.5	48 (9.9)	13 (2.7)	17 (3.5)	4 (0.8)	82 (16.8)
8	0.6	59 (12.1)	25 (5.1)	7 (1.4)	1 (0.2)	92 (18.9)
9	0.6	63 (12.9)	38 (7.8)	8 (1.6)	2 (0.4)	111 (22.8)
10	0.7	40 (8.2)	18 (3.7)	10 (2.1)	4 (0.8)	72 (14.8)
11	0.8	22 (4.5)	6 (1.2)	8 (1.6)	3 (0.6)	39 (8)
12	0.9	6 (1.2)	0	0	1 (0.2)	7 (1.4)
14	1	1 (0.2)	0	0	0	1 (0.2)

* The total number of antimicrobial agents used was fourteen. Antimicrobial resistant pattern for one and thirteen is absent. Figures in parentheses indicate percentages. MAR=Multiple antibiotic resistance, APEC=Avian pathogenic *Escherichia coli*

**Figure-4:**
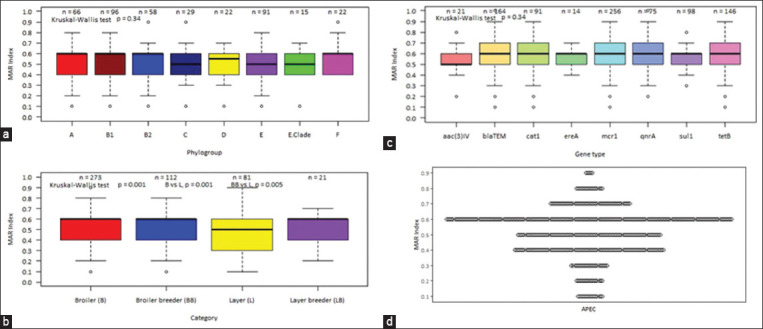
Genotype-phenotype association. (a) Box plot showing the comparison of the MAR index of isolates from different phylogenetic groups (b) Boxplots showing the comparison of MAR index with various types of chickens (c) Boxplots showing the relation between MAR index of an isolate and the detection of different gene by polymerase chain reaction (d) Scatter plot showing the MAR index of avian pathogenic *Escherichia coli* isolates; dots represent each isolate’s response to show variability and distribution of data. MAR=Multiple antibiotic resistance. *blaTE*M=Resistant gene of beta-lactam, *sul1*=Resistant gene of sulphonamides*, qnr*A=Resistant gene of quinolone, *tetB*=Resistant gene of tetracyclin*e, cat1*=Resistant gene of chloramphenico*l*, *ereA=*Resistant gene of erythromycin, *mcr1*=Resistant gene of colisti*n, aac(3)-IV*=Resistant gene of gentamicin, APEC = Avian pathogenic *Escherichia coli*.

Almost all APEC isolates (99%, n = 482) showed a MAR index above 0.2 (Table-4 and Figure-4). Overall, the maximum number of isolates R to eight and nine antimicrobial agents with a MAR index of 0.6 (41.7%, n = 203) was followed by seven antimicrobial agents with a MAR index of 0.5 (16.8%, n = 82). Based on the MAR index, a very low number of isolates (0.2%, n = 1) had the highest MAR index (Table-4).

**Figure-5:**
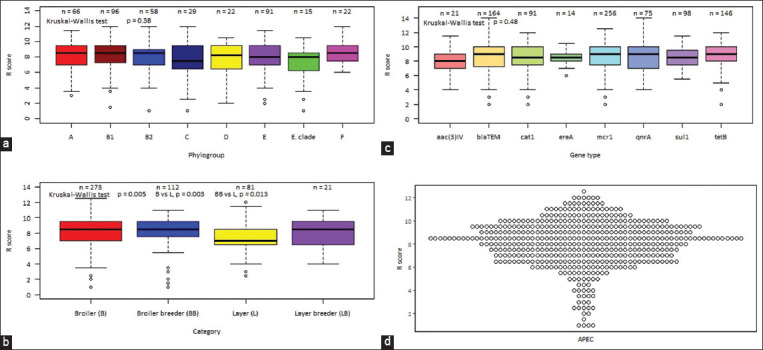
Genotype-phenotype association. (a) Box plot showing the comparison of the R-score of isolates from different phylogenetic groups (b) Boxplots showing the comparison of R-score with various types of chickens (c) Boxplots showing the relation between R-score of an isolate and the detection of a different gene by PCR (d) Scatter plot showing the R-score of APEC isolates; dots represent each isolate’s response to show variability and distribution of data. R-score=resistance score. *blaTE*M=Resistant gene of beta-lactam, *sul1*=Resistant gene of sulphonamides*, qnrA*=Resistant gene of quinolone, *tetB*=Resistant gene of tetracyclin*e, cat1*=Resistant gene of chloramphenico*l*, *ereA=*Resistant gene of erythromyci*n, mcr1*=Resistant gene of colistin, *aac(3)-IV*=Resistant gene of gentamicin, APEC=Avian pathogenic *Escherichia coli*, PCR=Polymerase chain reaction.

To test whether the types of chicken had any effect on their level of resistance, their MAR index and R-score were compared. Overall, the lowest MAR index and R-score were observed in the samples identified from the layer, and a significant difference among chicken types was found (p = 0.000, Figures-4 and 5). The MAR index and R-score were significantly different between broiler and layers, broiler breeder, and layers but not with layer breeder.

In total, 91.6% of APEC isolates (n = 446) were MDR from all chicken types (Table-5). Among the eight classes of antimicrobial agents, the highest number of isolates was MDR to four classes of antimicrobial agents (39.8%, n = 194, Table-5) followed by five classes of antimicrobial agents (29%).

**Table-5:** Distribution of MDR profile of APEC isolates (n = 487) in commercial chickens.

Number of antimicrobial classes	MDR	Broiler	Broiler breeder	Layer	Layer breeder	Overall
1	–	6 (1.2)	3 (0.6)	3 (0.6)	0	12 (2.5)
2	–	10 (2.1)	3 (0.6)	11 (2.3)	5 (1.0)	29 (6)
3	+	56 (11.5)	20 (4.1)	20 (4.1)	4 (0.8)	100 (20.5)
4	+	111 (22.8)	49 (10.1)	29 (6)	5 (1)	194 (39.8)
5	+	84 (17.2)	33 (6.8)	18 (3.7)	6 (1.2)	141 (29)
6	+	5 (1)	4 (0.8)	0	1 (0.2)	10 (2.1)
7	+	1 (0.2)	0	0	0	1 (0.2)

Figures in parentheses indicate percentages. APEC isolates resistant to all eight groups is absent. The total number of antimicrobial classes used was eight. APEC=Avian pathogenic *Escherichia coli*, MDR=Multidrug resistance

### ARGs and their patterns

**Figure-6:**
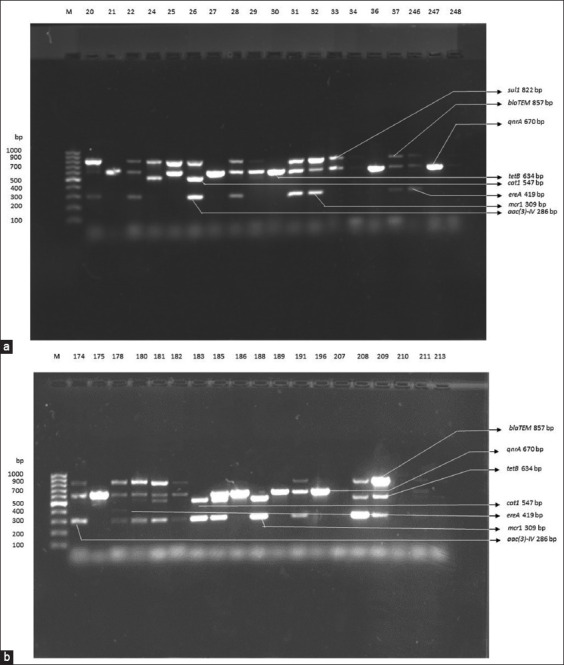
(a) PCR amplification for the detection of ARGs. (Lane M: 100 bp band [Marker], Lane 2 – 19 [Sample no 20 – 248] positive DNA [*blaTEM* 857 bp, *sul1* 822 bp, *qnrA* 670 bp, *tetB* 634 bp, *catA1* 547 bp, *ereA* 419 bp, *mcr*1 309 bp, *aac(3)-IV* 286 bp]). (b) PCR amplification for the detection of ARGs. (Lane M: 100 bp band [Marker], Lane 2 – 19 [Sample no 174 – 213] positive DNA [*blaTEM* 857 bp, *sul1* 822 bp, *qnrA* 670 bp, *tetB* 634 bp, *catA1* 547 bp, *ereA* 419 bp, *mcr*1 309 bp, *aac(3)-IV* 286 bp]). PCR=Polymerase chain reaction, ARGs=Antibiotic resistance genes, *blaTE*M=Resistant gene of beta-lactam, *sul*1=Resistant gene of sulphonamides*, qnrA*=Resistant gene of quinolone, *tetB=*Resistant gene of tetracycline*, catA*=Resistant gene of chloramphenicol, *ereA*=Resistant gene of erythromyci*n, mcr1*=Resistant gene of colistin*, aac(3)-IV*=Resistant gene of gentamicin, DNA=Deoxyribonucleic acid.

A minimum of one ARG was identified in 439 (90.1%) isolates (Figures-6a and b, Table-6). Among all the isolates, 48 (9.9%) exhibited a complete absence of ARGs. Eight distinct resistance genes were identified (Table-6). The most prevalent gene was the *mcr1* found in 256 (52.6%) isolates. Following closely, the *blaTEM* was the next most common, identified in 164 (33.7%) (Table-6, Figures-6a and b). The *ereA* was the least detected, present in 14 (2.9%) of all APEC isolates. In particular, the detection of *blaTEM*, *tetB*, *sul1*, and *mcr1* (in broiler) were highly significant (p < 0.001, Table-6) compared to other tested genes. Likewise, the detection of *cat1*, *aac(3)-IV* (in broiler), and *ereA* (in layer) were significant (p < 0.05, Table-6) compared to other tested genes.

**Table-6:** Distribution of ARGs in APEC strains from different types of commercial chickens.

ARGs	Broiler	Broiler breeder	Layer	Layer breeder	Overall	Chi-square value	p-value
*blaTEM*	65 (13.3)	49 (10.1)	45 (9.2)	5 (1)	164 (33.7)	35.264**	<0.001
*sul1*	87 (17.9)	0	10 (2.1)	1 (0.2)	98 (20.1)	57.776**	<0.001
*qnrA*	39 (8.0)	14 (2.9)	19 (3.9)	3 (0.6)	75 (15.4)	5.039	0.169
*tetB*	85 (17.5)	47 (9.7)	10 (2.1)	4 (0.8)	146 (30)	21.031**	<0.001
*cat1*	35 (7.2)	30 (6.2)	21 (4.3)	5 (1)	91 (18.7)	14.175*	0.003
*ereA*	4 (0.8)	4 (0.8)	6 (1.2)	0	14 (2.9)	8.719*	0.033
*mcr1*	116 (23.8)	78 (16)	52 (10.7)	10 (2.1)	256 (52.6)	28.814	
*aac(3)-IV*	13 (2.7)	0	6 (1.2)	2 (0.4)	21 (4.3)	8.444*	0.038

Figures in parentheses indicate percentages.

* and

** indicates significant (p ≤ 0.05) and highly significant (p ≤ 0.01), respectively. APEC=Avian pathogenic *Escherichia coli*, *blaTEM*=Resistant gene of beta-lactam, *sul1=*resistant gene of sulphonamides, *qnrA*=Resistant gene of quinolone, *tetB=R*esistant gene of tetracycline*, cat1*=*R*esistant gene of chloramphenicol*,* *ereA=R*esistant gene of erythromycin*, mcr1=R*esistant gene of colistin*, aac(3)-IV=*Resistant gene of gentamicin, ARG=Antibiotic resistance gene

Among the APEC isolates, five ARG patterns were identified. The most prevalent pattern, found in 35 (6.3%) of isolates, was *blaTEM–qnrA–mcr1*, followed by *cat1–mcr1* present in 31 isolates (5.1%) (Table-7). In terms of number of ARGs, one AMR gene was detected in 167 (34.3%) of the APEC isolates examined, followed by two genes in 135 (27.7%), three genes in 120 (24.6%) and four genes in 17 (3.5%). The highest MDR was observed in APEC isolates with one ARG (24.8%), followed by those with three ARGs (16.8%) (Table-7).

**Table-7:** Most common ARG patterns in APEC isolated from commercial chickens (n = 487).

Pattern	Resistance gene pattern	ARGs pattern (No.)	Total No.	MDR isolates	Overall MDR isolates
0 gene		48	48 (9.9)	38	38 (7.8)
1 gene	*aac(3)–IV*	5	167 (34.3)	4	121 (24.8)
	*blaTEM*	25		17	
	*cat1*	5		3	
	*ereA*	6		6	
	*mcr1*	51		38	
	*qnrA*	7		4	
	*sul1*	9		8	
	*tetB*	59		41	
2 genes	*blaTEM–mcr1*	17	135 (27.7)	9	71 (14.6)
	*blaTEM–qnrA*	16		10	
	*blaTEM–sul1*	3		2	
	*blaTEM–tetB*	5		4	
	*cat1–mcr1*	31		17	
	*qnrA–mcr1*	14		7	
	*sul1–cat1*	9		4	
	*sul1–mcr1*	4		3	
	*sul1–tetB*	18		10	
	*tetB–mcr1*	11		5	
3 gene	*tetB–cat1–mcr1*	5	120 (24.6)	5	82 (16.8)
	*blaTEM–cat1–mcr1*	17		10	
	*blaTEM–qnrA–mcr1*	35		22	
	*blaTEM–sul1–mcr1*	10		8	
	*blaTEM–tetB–aac(3) IV*	3		1	
	*blaTEM–tetB–mcr1*	18		11	
	*sul1–cat1–aac(3) IV*	3		3	
	*sul1–cat1–mcr1*	8		7	
	*sul1–qnrA–mcr1*	3		3	
	*sul1–tetB–mcr1*	17		12	
4 gene	*blaTEM –sul1–cat1–mcr1*	4	17 (3.5)	2	8 (1.6)
	*blaTEM –tetB–cat1–mcr1*	10		3	
	*sul1–cat1–ereA–aac(3)–IV*	3		3	

The figure in parenthesis indicates the percentage of isolates with each resistance gene pattern. ARG=antibiotic resistance gene, APEC=avian pathogenic *E. coli*, MDR=Multidrug resistant, *blaTEM*=Resistant gene of beta-lactam, *sul1=*Resistant gene of sulphonamides, *qnrA=*Resistant gene of quinolone, *tetB=R*esistant gene of tetracycline*, cat1=R*esistant gene of chloramphenicol*,* *ereA=R*esistant gene of erythromycin*, mcr1=R*esistant gene of colistin*, aac(3)-IV=*Resistant gene of gentamicin

### Pairwise correlation among different antimicrobials

**Figure-7:**
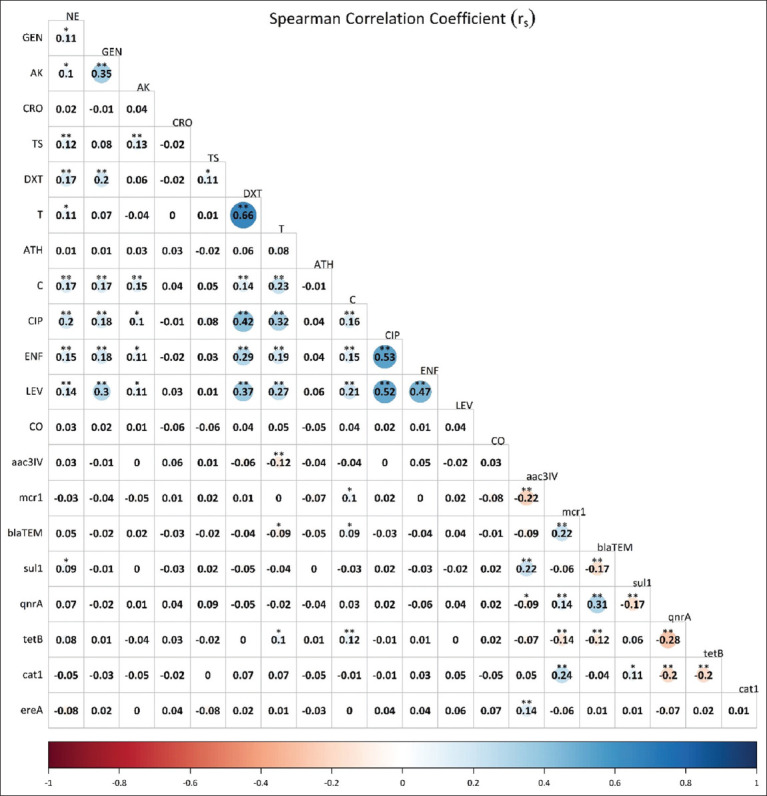
The genotypic and phenotypic antimicrobial resistance correlation. A pseudo-colored asymmetric correlation matrix representing the correlation between each pair of antibiotics according to pattern of susceptibility of all 487 APEC isolates from different types of chickens. Spearman’s rank correlation coefficient (r_s_) are shown for each antibiotic’s pairs. The corrPlot R package and the basic hclust function were used to create this figure. **and *resemble 1% (p-value ≤ 0.01) and 5% (p-value ≤ 0.05) level of significance, respectively, *blaTE*M=Resistant gene of beta-lactam, *sul1*=Resistant gene of sulphonamides*, qnrA*=Resistant gene of quinolone, *tetB*=Resistant gene of tetracyclin*e, cat1*=Resistant gene of chloramphenico*l*, *ereA=*Resistant gene of erythromyci*n, mcr1*=Resistant gene of colisti*n, aac(3)-IV*=Resistant gene of gentamicin, NE=Neomycin, GEN=Gentamicin, AK=Amikacin, AP=Ampicillin, CRO=Ceftriaxone, TS=Cotrimoxazole, DXT Doxycycline, T=Tetracycline, ATH=Azithromycin, C=Chloramphenicol, CIP=Ciprofloxacin, ENF=Enrofloxacin, LE= Levofloxacin, CO=Colistin, APEC=avian pathogenic *Escherichia coli*.

In our analysis, we observed significant correlation among phenotypic resistance to various antibiotics. T resistance showed strong correlation with DXT and C (r_s_ = 0.43**, 0.21**, respectively) and CIP (r_s_ = 0.20**), all highly significant (p < 0.001). In addition, a significant correlation was noted with AK (r_s_ = 0.11*, p ≤ 0.05, Figure-7). CIP, which had the second highest phenotypic resistance (Table-3), demonstrated significant correlations with LEV (r_s_ = 0.45**, Figure-7), GEN (r_s_ = 0.17**), and C (r_s_ = 0.14**), all highly significant (p < 0.001, Figure-7). Similarly, DXT phenotypic resistance was strongly correlated with C, TS, AK, and GEN (r_s_ = 0.20**, 0.16**, 0.15**, and 0.15**, respectively), all highly significant (p < 0.001), with a significant correlation with NE (p ≤ 0.05, Figure-7). Furthermore, LEV phenotypic resistance (Table-3) was correlated with C (r_s_ = 0.28**), DXT (r_s_ = 0.23**), GEN (r_s_ = 0.21**), and T (r_s_ = 0.22**), all highly significant (p < 0.001, Figure-7). NE phenotypic resistance was found to be significantly correlated with C, ENF, AK, and TS (r_s_ = 0.20**, 0.21**, 0.14**, and 0.12**, respectively, p < 0.001, Figure-7). ENF, with the highest I (Table-3), was correlated with CIP (r_s_ = 0.23**), LEV (r_s_ = 0.21**), DXT (r_s_ = 0.18**), and T (r_s_ = 0.15**), all highly significant (p < 0.001, Figure-7), with a significant correlation with GEN, AK, and C (p ≤ 0.05, Figure-7). C phenotypic resistance correlation with GEN and AK (r_s_ = 0.16** each, Figure-7) was highly significant (p < 0.001). Finally, the pair of AK with TS was significantly (p ≤ 0.05, Figure-7) and negatively associated with AP and ATH.

In the pairwise correlation analysis between ARG and AMR phenotype, the highest prevalent *mcr1* (Table-6) was highly correlated with *cat1*, *blaTEM_,_* and *qnrA* (r_s_ = 0.24**, 0.22** and 0.14**, respectively). There was a significant negative correlation of *mcr1* with *aac(3)-IV*, and *tetB* (r_s_ = –0.23** and –0.14**, respectively), all highly significant (p < 0.001, Figure-7). The *blaTEM*, the second most prevalent (19.5%) (Table-6), was significantly associated with the *qnrA* (r_s_ = 0.31**); however, it was negatively correlated with the *sul1* and *tetB* (r_s_ = –0.17**, and –0.12**, respectively), all highly significant (p < 0.001, Figure-7). In our study, relatively, high prevalence of *tetB* was associated with high phenotypic resistance against C (45.4%), which is supported by a highly significant correlation (r_s_ = 0.16**) between the *tetB* and phenotypic resistance to *cat1*. The *tetB* was significantly negatively associated with *qnrA* and *cat1* (r_s_ = – 0.28**, – 0.20**, respectively) (p < 0.001, Figure-7). Similarly, the *sul1* showed genotypic correlation with *aac(3)-IV*, (r_s_ = 0.22**), and negative association with *qnrA* (r_s_ = –0.17**, p < 0.001, Figure-7) which was highly significant with *cat1* (r_s_ = 0.11*). The *qnrA* was negatively associated with *cat1* (r_s_ = –0.20**), highly significant (p < 0.001), and with the *aac(3)-IV*, significant (r_s_ = –0.09*, p ≤ 0.05, Figure-7). Likewise, the *ereA* with the lowest prevalent genotypic resistance (1.7%), highly significantly correlated with *aac(3)-IV* (r_s_= 0.14**, p < 0.001).

Comparison of phenotypic and genotypic AMR results showed that the *tetB* was a predictor of resistance to antibiotic agents (Odds ratio [OR]: 1.91, 95% Confidence interval [CI]: 1.04–3.68, p = 0.03) and the *mcr1* was a predictor of susceptibility to the antibiotic agent (OR: 0.35, 95% CI: 0.23–0.54, p < 0.001). Non-significant positive and negative associations were found with the remaining AMR phenotypes and genotypes (Table-8).

**Table-8:** Binary logistic regression analysis of AMR in APEC isolates according to phenotypic and genotypic resistance.

Antibiotic	NP	ARG	NG	P–G+	P+G–	P+G+	P–G–	Odds ratio	95% CI	p-value
NE, 10 μg	447 (91.8)	*aac(3)-IV*	21 (4.3)	1	427	20	21	1.83	0.28–77.61	0.56
GEN, 10 μg	245 (50.3)	*aac(3)–IV*	21 (4.3)	11	235	10	21	0.89	0.33–0.57	0.80
AK, 30 μg	73 (15)	*aac(3)-IV*	21 (4.3)	18	70	3	21	0.94	0.17–3.36	0.93
AP, 10 μg	487 (100)	*blaTEM*	164 (33.7)	0	323	164	164	0		
CRO, 5 μg	183 (37.6)	*blaTEM*	164 (33.7)	106	125	58	164	0.87	0.57–1.30	0.47
TS, 25 μg	316 (64.9)	*sul1*	98 (20.1)	33	251	65	98	1.08	0.66–1.79	0.78
DXT, 30 μg	438 (89.9)	*tetB*	146 (30)	15	307	131	146	0.97	0.49–1.98	0.92
T, 30 μg	406 (83.4)	*tetB*	146 (30)	16	276	130	146	1.91	1.04–3.68	0.03
ATH, 15 μg	15 (3.1)	*ereA*	14 (2.9)	14	15	0	14	0	0–10.27	1
C, 30 μg	210 (43.1)	*cat1*	91 (18.7))	53	172	38	91	0.93	0.57–1.52	0.77
CIP, 5 μg	462 (94.9)	*qnrA*	75 (15.4)	3	390	72	75	1.35	0.39–7.24	0.63
ENF, 30 μg	458 (94)	*qnrA*	75 (15.4)	7	390	68	75	0.55	0.22–1.58	0.18
LEV, 5 μg	434 (89.1)	*qnrA*	75 (15.4)	6	365	69	75	1.48	0.59–4.40	0.38
CO, 0.8 mg (MIC value)	156 (32)	*mcr1*	256 (52.6)	183	83	73	256	0.35	0.23–0.54	<0.001

Figures in parentheses indicate percentages. NP=Number of APEC isolates expressing phenotypic resistance, ARG=Antibiotic resistance gene, NG=Number of APEC isolates carrying the indicated resistance gene, P+/G+=Number of phenotypically resistant APEC isolates (P+) with resistance gene (G+) for the drug identified, P+/G–=number of phenotypically resistant APEC isolates (P+) with no resistance gene (G–) for the drug identified, P–/G+=Number of phenotypically susceptible APEC isolates (P–) with resistance gene (G–) for the drug identified, P–/G–=Number of phenotypically susceptible APEC isolates (P–) with no resistance gene (G–) for the drug identified, CI=Confidence interval. OR > 1 means that the resistance gene is introduced as a predictor of resistance to antibiotic agents, but OR < 1 means that the resistance gene is a predictor of susceptibility to antibiotic agents. APEC=Avian pathogenic *Escherichia coli*, AMR=Antimicrobial resistance, *blaTEM*=Resistant gene of beta-lactam, *sul1*=Resistant gene of sulphonamides, *qnrA*=Resistant gene of quinolone, *tetB=*Resistant gene of tetracycline*, cat1=*Resistant gene of chloramphenicol*,* *ereA=*Resistant gene of erythromycin*, mcr1=*Resistant gene of colistin*, aac(3)-IV=*Resistant gene of gentamicin, NE=Neomycin, GEN=Gentamicin, AK=Amikacin, AP=Ampicillin, CRO=Ceftriaxone, TS=Cotrimoxazole, DXT=Doxycycline, T=Tetracycline, ATH=Azithromycin, C=Chloramphenicol, CIP=Ciprofloxacin, ENF=Enrofloxacin, LEV=Levofloxacin, CO=Colistin, MIC=Minimum inhibitory concentration, bp=base pair

## Discussion

The primary challenge in routine AMR surveillance lies in the concurrent detection of both genotypic and phenotypic resistance. This study in Nepal sheds light on the limitation of relying on phenotypic resistance, as it does not consistently align with genotypic resistance. This is the first study in Nepal to comprehensively characterize antibiotic susceptibility pattern, MDR profiles, their correlation, and the molecular identification of ARGs in APEC obtained from colibacillosis-infected broiler, broiler breeder, layer, and layer breeder chickens. It is interesting to note that most APEC isolates exhibited MDR along with presence of ARGs, with the *mcr1* identified as the most prevalent gene. Intriguingly, despite the prevalence of the *mcr1*, the CO itself demonstrated high phenotypic sensitivity. This underscores the necessity of monitoring both phenotypic and genotypic resistance is necessary to accurately evaluate the AMR landscape in a particular area.

Our study unveiled diverse antimicrobial sensitivity and resistance patterns across different chicken types investigated. Notably, it was observed that none of the antibiotics were 100% effective except for CO in layer breeders. Overall, 100% resistance was observed to AP and more than 90% resistance to CIP and ENF in isolates from all chicken types. We also found a significant correlation among the most R antimicrobials in our study. However, we found decreased resistance rates to GEN, AK, TS, T, and C, as reported by Bista *et al*. [41]. While other studies reported higher resistance percentages against cotrimoxazole [41–43], AK, C, T, and GEN [41], our study identified similar resistance percentages against AK [42–44], CIP, and LEV [41, 44]. These studies revealed that APEC isolates were highly S to AK, ATH, and CO. These collective observations suggest a positive trend in antibiotic reduction, potentially indicative of improved antibiotic stewardship due to government-imposed restrictions.

We found 99.4% AP resistance in *E. coli* across various types of chickens; however, the phenotypic resistance did not correlate with genotypic resistance in this study. Previous studies have reported high rates of resistance to AP globally, with figures such as 98% in Nepal [43], 96.3% in Qatar [45], 80% in Iran [46], and 97.3% in Egypt [47]. In contrast, all MDR APEC isolated from broiler chickens were completely R to AP and T in Nepal [42], Bangladesh [4], Qatar [45], and China [48]. T, a highly R antibiotic, showed a significant correlation with phenotypic resistance to C, CIP, ENF, and LEV. In contrast with our observation, the higher resistance rate of T reported in earlier studies (93%–100%) [4, 11, 41, 47–49], Johar *et al*. [45] in Qatar reported a similar prevalence to this study. However, lower resistance was reported in Jordan than in our study (65%) [50]. Among the top seven antibiotics used, AP, amoxicillin, CRO, and GEN are frequently misused [9]. The observed high resistance to AP and T in this study could be attributable to the fact that T is the most consumed antimicrobial and AP is the most inappropriately prescribed antimicrobial in broiler and layer farms in Nepal [9].

We identified a CIP resistance rate of 82.5%, a figure similar to that reported by Bista *et al*. [41], although it was higher than the resistance rate (48%–75%) reported in other studies [4, 44, 46, 48–51]. In contrast, a higher resistance rate 97.8% was reported by Johar *et al*. [45]. These discrepancies may be due to the overuse of CIP for the treatment of infectious diseases in poultry.

Subsequently, 67.1 % of APEC isolates were DXT R, which is lower than the resistance rate of 87.5% reported by Sankhi et al. [44] in Nepal, 92.2% [50] in Jordan, 100% [48] in China). However, it surpassed the 46.6% and 68.1% resistance rates reported by Chapagain and Thapa [42] and Bista *et al*. [41], respectively, in a similar study conducted in Nepal.

In the present study, 76.4% of the APEC isolates were R to LEV, which is higher than the resistance rate 45.8% [42] and 72.5% [44] reported in Nepal. Comparable resistance rates of 76.4% in Nepal [41] and 81.7% in India [49] were also reported.

Furthermore, we observed 56.3% resistance rate to TS, among the APEC isolates, a percentage lower than the 86.4% resistance rate reported by Chapagain and Thapa in Nepal [42], 82.2% in Egypt [47], 68.5% in Iran [46], and 95.5% in Jordan [50]. Similar resistance rates of 68.1% and 56.7% were reported by Bista *et al*. [41] and Limbachiya *et al*. [49], respectively, both surpassing the lower resistance rates 16.6%–18.1% reported [48, 52].

In addition, 47% of the isolates were R to NE, which is significantly higher than the resistance rate of 12.5% reported in China [48]. This high rate of resistance is likely due to the broader use of NE in poultry without prescription as a common practice in Nepal [9].

In the present study, 97.4% of the APEC isolates were I to ENF, which is concerning for the clinical drug use. The resistance rates (84.4% and 55.5%) reported by Ievy *et al*. [4] and Ibrahim *et al*. [50], respectively, are relatively lower than those in our study. Due to excessive or incorrect use of ENF in Nepal, a high resistance or I has developed.

In the current study, 32.9% of the APEC isolates were chloramphenicol R, which is lower than the resistance rates 58.3% reported by Bista *et al*. [41] in Nepal, 97.2% in Bangladesh [4], and 70% in India [49].

We found that 31% of the APEC isolates had phenotypic antibiotic resistance to GEN, which is less than the resistance rates of 76.4% reported in Nepal by Bista *et al*. [41], 76.7% in India [49], 57.2% in Jordan [50], 43.8% in Egypt [47], and 68.2% in China [48]; however, a similar resistance rate 32.5% was reported by Sankhi *et al*. [44] from Nepal. Lower resistance rates of 8.3% and 10.4% were reported from other parts of the world [4, 45], which were less common. The pair of GEN and AK is significantly associated in terms of phenotypic resistance.

In the present study, only 25.7% of the APEC isolates were R to CRO, which is lower than the 91% resistance rate reported in Canada [53] and 46% in Nepal [41]. Only 7.2% of the APEC isolates were Rto AK. In contrast, a high resistance rate of 71.5% was reported by Bista *et al*. [41] in Nepal and 80% in India [49]. Similar to our findings, a low resistance rate of AK [48] was found and a slightly higher resistance rate (11%–17.5%) was reported [42–44] in Nepal. The limited use of CRO and AK is primarily because of its availability only in parenteral form. In addition, its infrequent use in poultry disease treatment might have consequently resulted in lower resistance rates.

In our study, 4.7% APEC isolates were R to CO. In contrast, high phenotypic resistance (100%) to CO was detected among APEC isolates from broiler chickens in India [49] and 33% in Qatar [45] which might be related to the use of CO on chicken farms as a therapeutic drug. In contrast to another study from Nepal [41, 54] and Egypt [55], we found very low phenotypic resistance with a very low MIC 0.98 μg, which could be due to the ban of CO. CO should not be used for animal production because it is a last-resort antibiotic for human use. The Ministry of Agriculture and Livestock Development of Nepal has announced that there are no antibiotics in feed, and further monitoring and evaluation are needed. This could be the reason behind the reduced resistance compared with previous studies in Nepal.

However, none of the APEC isolates were susceptible to most of the VCIA in different studies [41, 43, 49, 53], which is similar to our findings. The most concerning result of this study was the high percentage of APEC isolates from various types of chickens that were R (21%–99.2%) to most first-line therapeutic antibiotics used to treat poultry disease, except for AK, ATH, and the antibiotic used as a last resort, CO (7.2%, 3.1%, and 4.7%, respectively). Recently, published Nepal Government’s report mentioned resistance of poultry to commonly prescribed antibiotics [56]. There is an extreme regular abuse of antibiotics in poultry feed and drinking water as feed additives to improve efficiency and weight gain [57] and to minimize production losses in the case of high mortality in Nepal [9]. Numerous antibiotics have been indiscriminately used to treat colibacillosis, especially in low-middle-income nations such as Nepal, where the use of antibiotics results in selective pressure for the emergence of drug-R APEC and contributes to the growth and spread of AMR, posing significant problems for public health [58]. The AMR also has an impact on the sustainable development goals, particularly those that address hunger, poverty, malnutrition, health, and economic growth [59]. Multiple antibiotics, including the most R AP and tetracycline, were used for the treatment and prevention of bacterial poultry disease at suboptimal levels, which may be the main cause of the high resistance rates in veterinary practice in Nepal [60]. The previous study also supports the marked increase in veterinary antibiotic sales in Nepal [61] in the past few years. In most previous studies, samples were taken from dead chickens infected with colibacillosis, but the use of antibiotics without AST may have resulted in the development of MDR isolates. In addition, cross-resistance among antibiotics can also contribute to the observed high resistance, as a significant correlation with a p < 0.001 was detected in this study.

Overall, the AST pattern was inconsistent due to variations in the protocols, maintenance of standard turbidity for inoculum, and antibiotic disks from different companies having different potency. The disk diffusion technique, despite being the most used approach in Nepal and other parts of the world for determining AMR because of its simplicity, effectiveness, and low cost, has significant drawbacks [62]. AMR detection of CO by the disk diffusion test is completely incorrect. The antibiotic disk quality for AMR using the disk diffusion method is one of the major factors for variation in AST reports. The antibiotic disks available in the market in Nepal had low efficacy and considerable variance in the zone of inhibition, which led to variation in the AMR profile [63]. They were unable to achieve MIC values and were labor-intensive and time-consuming. These results may be more surprising [23] than broth microdilution. HiMedia, a manufacturer of low-potency disks, exhibited significant quality issues with 33% of readings being outside the normal range and surprising variations in inhibition zone sizes (4–9 mm) for disks contained in the same vial [63]. In this study, phenotypic detection of CO AMR was performed by BMD, and the detection of ARGs from APEC isolates was performed by multiplex PCR, which is the most reliable and sensitive test. The tested panel of antimicrobials exhibited the lowest levels of resistance, which is probably a result of the fact that Nepal uses less or no antimicrobials on poultry as well as there was a lack of correlation between genotype and phenotype in our investigation. A significantly lower phenotypic resistance rate observed for CO in our study than in other studies [41] is due to the breakpoint concentration for resistance being 4 μg/mL used instead of 2 μg/mL according to EUCAST [34] and in our study. The weak correlation among different antimicrobials (Figure-7) indicates a need of monitoring genotypic ARGs more often than those phenotypic AMR profiles.

This study’s MAR index value was larger than 0.2, which is very high (99%) in a similar study [41, 49, 64], indicating high-risk sources of contamination where various antibiotics may frequently be used for disease prevention. This MDR raises major concerns about the indiscriminate abuse of numerous antibiotics for both prevention and treatment, which would remove the drug-sensitive APEC from poultry industry where antibiotics are abundant. To test whether the types of chicken had any effect on their level of resistance, their MAR index and R-score were compared. Overall, the lowest MAR index and R-score were observed in the samples identified from the layer, and a significant difference was found among chicken types. This indicates that less antibiotics are used in layers compared with other types of chickens. The MAR index and R-score were significantly different between broiler and layers and between broiler breeder and layers but not between layer breeder. Overall, a non-significant difference was observed among the phylogenetic group and ARGs of the APEC isolates for the MAR index and R-score on the level of resistance. The lowest overall MAR index and R-score were observed in the *Escherichia* clade phylogroup and *aac(3)-IV* of aminoglycoside resistance, respectively.

MDR bacteria are a growing clinical problem for both public health and the poultry industry. This study reported a significantly very high percentage of MDR (91.6%) in APEC isolates from different chicken types, and 73.3% of the isolates showed resistance against 7–10 different antimicrobials. Several national and international studies reported very high MDR isolates (80%–100%), which are similar to our findings from chicken [23, 43, 45], indicating that there is a high persistence of MDR organisms in our food chain. In alignment, previously detected high MDR-APEC prevalence (100%) in other food chain studies in migratory birds [65] are strong evidence of possible transmission of MDR organisms in our food chain system, which is perilous. However, the alarming finding from our study is that most of the APEC isolates were MDR in class, which renders treatment options with antibiotics. Overall, it is hypothesized that an increase in antibiotic resistance increases the likelihood of recurrent infection [66], causes hundreds of millions of deaths in humans [19], declines in livestock production resulting in enormous financial loss [67], overtakes cancer as the top cause of death worldwide by 2050 [68], and may lead these pathogens into untreatable superbugs by 2050. Therefore, careful antibiotic use is strongly advised because overuse promotes bacterial populations to develop resistance.

Our study raises serious concerns about the elevated ARGs found in commercial chickens infected with APEC. ARGs were also found in APEC isolates from broiler chickens from other areas of Nepal [9, 41, 69]. The spread of APEC causing CO resistance and MDR in Nepal’s poultry farms has negative effects on consumers and poultry handlers. Because APEC is now confirmed to be a member of ExPEC groups, human infection may be possible [70]. All these findings demonstrate that the misuse of antibiotics in the poultry sector is highly concerning. The fact that the *mcr1* present in 52.6% of the isolates, even though only 4.7% of them had phenotypic resistance, was the most prevalent among the eight ARGs is a major concern nowadays. However, there was an inverse connection between phenotype and different genotypes, indicating a possible association between the emergence of ARGs and antimicrobials. A similar insight was mentioned by Varga *et al*. [52], who reported a lower prevalence of the *mcr1 (*1.2%) with all isolates phenotypically R to CO [71]. Since the discovery of *mcr1* in swine *E. coli* obtained from China [39], the relatively high *mcr1* have been detected in several other studies of APEC isolates from broilers (43.9% [41], 22.8% [55] in Nepal, 15.3% [72] in China, and 64.3% [53] in Canada. The constant use of CO on poultry farms may be responsible for this alarming finding of a high percentage of *mcr1*. One of the main reservoirs and transmitters of CO resistance is poultry [73]. According to va den Bogaard and Stobberingh [74], a high incidence of MDR strains of *E. coli* and other enterobacterial is related to the indiscriminate use of antibiotics. Our findings of MDR and CO-R APEC revealed substantial health risks to farm workers. However, more sophisticated research is needed to understand the intensity of the risks.

Plasmid-mediated beta-lactamases, *blaTEM*, were identified in the APEC isolates at a prevalence of 33.7%, indicating a problematic situation, as these genes are plasmid-borne and can be transferred to other bacteria, environments, and humans [75], making the latest generation cephalosporin ineffective. This differs from the findings of Rehman *et al*. [53] in Canada, who assessed the prevalence of *blaTEM*-producing *E. coli* (59.6%) in broilers and people living or working with broiler farms. According to a review of the transmission of ESBL-producing bacteria through products of livestock origin, chicken is a significant source of infection [76]. A very high prevalence of *blaTEM* (72%–100%) has been reported in several studies [11, 50, 72], except for a lower resistance (4.2%) from retail chicken [77]. However, some studies reported a complete absence of the *blaTEM* in commercial chicken [40, 49, 78, 79]. Most third-generation cephalosporins such as CRO are not used in the poultry industry in Nepal, and; they are used only by the parental route for the treatment of animal diseases such as mastitis in cattle. The significantly high percentage of *blaTEM* detection was highly significantly correlated with the most prevalent ARG *mcr1* in our study. This is a very serious issue because the possibility of ESBL resistance is highly associated with genotypic resistance to the last resort of the drug CO. Proper monitoring and control plans must be implemented to decrease the high percentage of *mcr1* and *blaTEM* in Nepal’s poultry production chain.

A *tetB* was prevalent in 30%, which is lower than previous studies, which found 30%–50% [46, 49] prevalence in APEC isolates. The APEC isolates had 20.1% *sul1*, which is lower than the prevalence of 28%–34% identified in APEC in a prior study in Iran and Canada [11, 40, 46, 49, 53]. The relatively high resistance of *sul1* (72.4%) was reported by Ibrahim *et al*. [50]. The relatively high prevalence of *sul1* (20.1%) was correlated with high phenotypic resistance against *cat1* (45.4%) in this study.

The results of the present analysis of *cat1* (18.7%) do not agree with those of other studies [49, 50, 53], which reported *cat1* in 20%–62% of the cases, whereas Shehata *et al*. [79] observed no prevalence of *cat1*. The prevalence of the *cat1* was highly significantly (p < 0.001) correlated with the highest resistance of the *mcr1* (52.6%) in our study. Both are mediated by plasmids and are the primary vectors for horizontal gene transfer of ARGs. This indicated a risk of transmission of MDR strains and ARGs in the food chain.

The current *qnrA* prevalence (15.4%) was similar to that reported in another study [11]. In contrast to previous findings, all isolates were negative for the *qnrA* [49], whereas Momtaz *et al*. [80] and Ponce-Rivas *et al*. [81] identified a high prevalence of this gene, up to 36.8% and 52.6%, respectively.

The relatively high presence of genes in particular isolates is significantly associated with high resistance to AP and T, which may be due to the presence of other genes that are not studied here. Similar AMR occurrences have also been documented [82]. Because of the complexity of the molecular pathways underlying AMR [83], the phenotype or genotype may occasionally be unable to reliably anticipate the fate of the other. Therefore, the presence or absence of a particular gene associated with a given phenotype does not imply that a certain strain is R or S to a given agent [84]. The discrepancies in genotype and phenotype observed in our study could be explained by the fact that all resistance genes were not tested, some genes were not activated, or some isolates contained “silent gene cassettes.”

It is known that some antibiotics might cause resistance when improperly used. It may function as a co-selection marker for other antibiotics. This might also occur with drug classes that are not related at all. *cat1* is rarely used in the poultry industry [85]; however, approximately 45% of the isolates showed phenotypic resistance to *cat1* in the context of Nepal with *cat1* ARG detected with 18.7%. Unlike our study, 100% resistance to CO by disk diffusion and no resistance genes were found in another study [49]. This study found a non-significant weak association of *tetB* across *E. coli* isolates. O’Connor *et al*. [85] hypothesized that this may be caused by the incompatibility of plasmids containing the T resistance determinants. However, more research is needed to determine the connections between the ARGs and the likely association to antimicrobial exposure.

In this study, we found a positive correlation between phenotypic and genotypic resistance, as reported in several other studies [11, 66, 86], indicating the haphazard use of antibiotics and cross-contamination during farm practices. Genotype resistance to different antibiotics (*tetA*, *sul2*) alone can predict the phenotype with higher sensitivity and specificity (85%–100%) in Salmonella [87]. A similar study is needed in APEC to determine if this type of correlation exists for the prediction of AMR in the future.

Our findings indicated that first-line therapeutic antibiotics, such as CO, AP with β-lactam resistance, T, TS, and fluoroquinolone, had a high genotypic antibiotic resistance profile. The use of several antibiotics without an antibiogram was discovered through direct discussion with the proprietor of the chicken source farm during the study. This practical strategy exerted positive genetic pressure on commensal and pathogenic strains of *E. coli*, which resulted in the development of AMR [88]. AMR has emerged during the past 10 years as a significant global threat to economic growth, public health, and animal and human health [68].

Notably, ARGs isolated from various chicken species in this study have also been found in humans and wild animals in the past. Because of their zoonotic nature, chickens may serve as a possible reservoir for MDR APEC, which can then spread to humans from these animals [89]. These antibiotic-R APECs can become established in birds and spread continually among flock as well. Although the results indicated that APEC isolates possess several phenotypic and genotypic resistances, interpretation may nevertheless be difficult. First, there is no single resistance gene or set of such genes associated with APEC in different commercial chickens. Second, most APEC strains are MDR, with the resistance gene being the most prevalent with the lowest phenotypic resistance. Third, even if a strain possesses several resistance genes, they may not be active. In addition, the resistance genes analyzed were located on genetic mobile elements, which may allow them to transfer to other microorganisms.

The study’s sample source, sample size, antimicrobial, and number of genes evaluated for antibiotic resistance are both limited. Data on the genotype and phenotypic antibiotic resistance profiles of APEC strains are still insufficient. Conducting additional in-depth research targeting more antimicrobials, including the most recent antibiotics, encompasses a larger area with diverse samples, including those from various origins such as human isolates would provide comprehensive insights into the AMR patterns, prevalent ARGs, and other aspects related to pathogens from food-producing chickens. Molecular serotyping and genome sequencing are necessary to determine the diversity of APEC, which is essential for designing potential vaccines to control avian colibacillosis with further reduction in the AMR problem. Given the rapidly expanding poultry business and the widespread consumption of chicken meat in Nepal, additional research should be conducted to understand the dynamics and genetic variety of antibiotic-R APEC linked to chickens, poultry farms, and their environments. There is high potential of transmission of AMR in chicken APEC to wildlife and other food animals [13]. Similarly, environmental (e.g., water sources, animal habitats, and foods) contamination [83] has been alarming to public health as well. Therefore, comprehensive and well-designed study is needed for the prevention and control of avian colibacillosis. Unfortunately, such studies require more financial resources and are often beyond the reach of routine diagnostics. In this study, the current state of APEC AMR in commercial chickens is revealed for the first time in Nepal. This information is useful for the clinical management of disease as well as the creation of policies and guidelines to lower AMR in Nepal’s commercial production. Since MDR APEC are zoonotic problems, it is essential to screen the MDR APEC strains in farms, food chains, among food handlers, and farm employees routinely. Further, emphasis should be given to identify resistance genes in APEC, thereby safeguarding human health, too. In addition, stringent measures must be taken to reduce the risks associated with AMR. These include implementing a special program to control the use of antibiotics in poultry production, monitoring and evaluating the effectiveness of biosecurity measures, preventing the misuse of antibiotics, and adapting the one-health approach.

## Conclusion

Overall, the detection of AMR profiles (phenotypic antimicrobial susceptibility and resistance pattern, MDR, ARGs, and MAR index) for APEC in broiler, layer, and breeder chickens have significant associations and are interlinked with each other. The zone of inhibition, which is the measure of *in vitro* susceptibility, is different against tested antimicrobials in those birds, indicating differences in susceptibility among chicken types. Phenotypically, CIP, LEV, tetracycline, and DXT were the most R and ATH, CRO, and CO were the most susceptible antimicrobials. Most APEC strains were MDR with the CO resistance gene, *mcr1* being the most prevalent with the lowest phenotypic CO resistance. ARGs and phenotypic resistance alone cannot determine AMR due to its inverse relationship, indicating that these chickens were potential reservoirs for antibiotic-R APEC posing serious health hazards. Therefore, we advise careful use of antibiotics elsewhere because their overuse encourages bacterial populations to develop resistance. Our findings of AMR profiles, therefore, assist in lessening their harmful consequences.

## Data availability

The supplementary data can be available from the corresponding author on a reasonable request.

## Authors’ Contribution

RKB: Conception and design of the study, resources, sample collection, laboratory work, investigation and data curation, interpretation of data, statistical analysis, validation, formal analysis, and drafted the manuscript. HBB, IPD and BD: Analysis and interpretation of data, revised the manuscript critically, and supervised the study. All authors have read, reviewed, and approved the final manuscript.
